# 3α-Hy­droxy­tirucalla-8,24-dien-21-oic acid

**DOI:** 10.1107/S1600536811008956

**Published:** 2011-03-23

**Authors:** S. Yousuf, R. S. T. Kamdem, B. T. Ngadjui, P. Wafo, Hoong-Kun Fun

**Affiliations:** a H.E.J. Research Institute of Chemistry, International Center for Chemical and Biological Sciences, University of Karachi 75270, Pakistan; b Department of Chemistry, Higher Teachers Training College, University of Yaounde I, PO Box 48 Yaounde, Cameroon; c Department of Organic Chemistry, University of Yaounde I, PO Box 812 Yaounde, Cameroon; d X-ray Crystallography Unit, School of Physics, Universiti Sains Malaysia, 11800 USM, Penang, Malaysia

## Abstract

The title compound, C_30_H_48_O_3_, a triterpene isolated from the resin of *canarium schweinfurthiiand*, is an isomer of the previously reported triterpene 3α-hy­droxy­tirucalla-7,24-dien-21-oic acid [Mora *et al.* (2001 ▶). *Acta Cryst.* C**57**, 638–640], which crystallizes in the same trigonal space group. The title mol­ecule consists of four fused rings having chair, half-chair, half-chair and envelope conformations for rings *A*, *B*, *C* and *D*, respectively (steroid labelling). An intra­molecular C—H⋯O hydrogen bond generates an *S*(7) ring. In the crystal, mol­ecules are linked by O—H⋯O and C—H⋯O inter­actions, forming (001) sheets.

## Related literature

For the crystal structure of 3α-hy­droxy­tirucalla-7,24-diene-21-oic acid, see: Mora *et al.* (2001 ▶). For the biological activity of *canarium schweinfurthiiand*, see: Atawodi (2010 ▶); Dongmo *et al.* (2010 ▶) and for its botany, see: Tchiégang *et al.* (2001 ▶). For the stability of the temperature controller used in the data collection, see: Cosier & Glazer (1986 ▶).
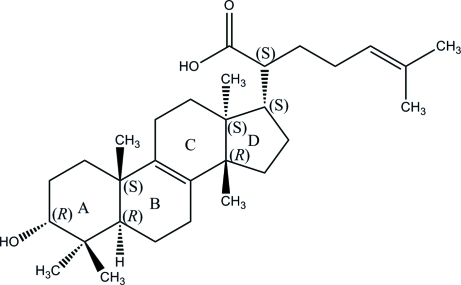


## Experimental

### 

#### Crystal data

C_30_H_48_O_3_
*M* *_r_* = 456.68
Trigonal, 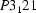

*a* = 11.2794 (2) Å
*c* = 36.6986 (6) Å
*V* = 4043.45 (12) Å^3^
*Z* = 6
Cu *K*α radiation
μ = 0.54 mm^−1^
*T* = 100 K
0.29 × 0.24 × 0.13 mm

#### Data collection

Bruker SMART APEXII DUO CCD diffractometer
Absorption correction: multi-scan (*SADABS*; Bruker, 2009) ▶ *T*_min_ = 0.859, *T*_max_ = 0.935
29022 measured reflections
5154 independent reflections
5062 reflections with *I* > 2σ(*I*)
*R*_int_ = 0.040

#### Refinement

*R*[*F* ^2^ > 2σ(*F* ^2^)] = 0.038
*wR*(*F* ^2^) = 0.104
*S* = 0.97
5154 reflections
305 parameters
H-atom parameters constrained
Δρ_max_ = 0.34 e Å^−3^
Δρ_min_ = −0.15 e Å^−3^
Absolute structure: Flack (1983 ▶), 2099 Friedel pairs
Flack parameter: −0.35 (19)

### 

Data collection: *APEX2* (Bruker, 2009 ▶); cell refinement: *SAINT* (Bruker, 2009 ▶); data reduction: *SAINT*; program(s) used to solve structure: *SHELXS97* (Sheldrick, 2008 ▶); program(s) used to refine structure: *SHELXL97* (Sheldrick, 2008 ▶); molecular graphics: *SHELXTL* (Sheldrick, 2008 ▶); software used to prepare material for publication: *SHELXTL* and *PLATON* (Spek, 2009 ▶).

## Supplementary Material

Crystal structure: contains datablocks global, I. DOI: 10.1107/S1600536811008956/hb5815sup1.cif

Structure factors: contains datablocks I. DOI: 10.1107/S1600536811008956/hb5815Isup2.hkl

Additional supplementary materials:  crystallographic information; 3D view; checkCIF report

## Figures and Tables

**Table 1:** Hydrogen-bond geometry (Å, °)

*D*—H⋯*A*	*D*—H	H⋯*A*	*D*⋯*A*	*D*—H⋯*A*
O1—H1*O*1⋯O2^i^	0.96	1.74	2.6762 (14)	162
O3—H1*O*3⋯O2^ii^	0.83	2.00	2.828 (2)	172
C12—H12*A*⋯O1	0.99	2.55	3.367 (2)	139
C22—H22*A*⋯O3^iii^	0.99	2.36	3.276 (2)	153

Symmetry codes: (i) 
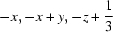
; (ii) 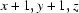; (iii) 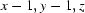.

**Fig. 1.**
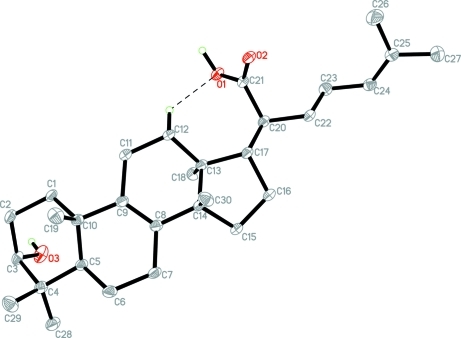
The molecular structure of the title compound, showing 30% probability displacement ellipsoids. The intramolecular hydrogen bond is shown by a dashed line.

**Fig. 2.**
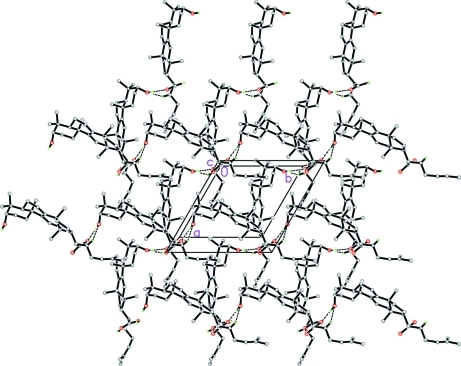
The crystal packing of the title compound, showing (001) sheets.

## supplementary crystallographic information

### Comment

3α-Hydroxytirucalla-8,24-dien-21-oic acid (or epielemadienolic acid) is a known triterpene derivative that was isolated from the resin of *canarium schweinfurthii*, a tree growing in equatorial forest regions from Cameroon, Central African Republic, Gabon to Congo (Tchiégang *et al.*, 2001). The plant is used for a variety of ailments including malaria, fever and diarrhea (Atawodi, 2010; Dongmo *et al.* (2010). As a part of our going research on the medicinaly important plants, the title compound was isolated from the dichloromethane soluble part and the structure was established on the basis of X-ray diffraction studies. The space group (P3_1_21) and cell parameters were found to be similar to the prevoiusly reported 3α-hydroxy-tirucalla-7,24-dien-21-oic acid (Mora *et al.*. 2001). However the bond lengths of C7-C8 [1.499 (2) Å] and C8-C9 [1.353 (2) Å] were found to be different from those reported for the previously reported triterpene [C7-C8 = 1.353 (2) Å and C8-C9 = 1.49 Å]. The difference in the bond lengths clearly indicates that the title compound is an isomer of previously reported 3α-hydroxy-tirucalla-7,24-dien-21-oic acid, having C8-C9 double-bond/olefin instead C7-C8 double-bond/olefin. The molecular structure showed that the trans fused rings A/B/C and D adopt chair [Q= 0.549 (2) Å, θ = 4.5 (2)° and φ = 51 (2)°], half-chair [Q = 0.545 (2) Å, θ = 50.0 (2)° and φ = 8.3 (3)°], half-chair [Q = 0.524 (19) Å, θ = 129.7 (2)° and φ = 63.6 (2)°] and envelope [Q = 0.487 (2) Å and φ = 13.5 (2)°] conformations respectively. The half-chair and envelope conformations of rings C & D are stabilized by C12—H12A···O1 intramolecular hydrogen bond. In the crystal structure, the molecules are linked to form infinite chains *via* O3—H1O3···O2, O1—H1O1···O2 and C24—H22A···O3 hydrogen bonds (symmetry codes as in Table 1) to form (001) sheets. (Fig. 2).

### Experimental

The resin (100 g) of *Canarium schweinfurthii* Engl. was collected in Yaounde, Cameroon in May 2010 and identified by Prof. Noumi, a botanist at the Department of Biology, University of Yaounde-1. A voucher specimen (HNC 25918.) was deposited at the National Herbarium of Cameroon in Yaounde. The resin (100 g) of *C. schweinfurthii* was allowed to dry under shade and extracted with dichloromethane. The extract (70 g) was subjected to column chromatography (CC) over silica gel (300 g, 60 × 5 cm) eluting with hexane follow by a mixture of n-hexane-EtOAc in order of increasing polarities. The fractions eluted were monitored by thin layer chromatography and similar fraction were combined to give seven fraction FrA-FrG. Fraction FrE was further subjected to purification on silica gel CC and eluted with mixture of hexane-acetone in a gradient to yield the title compound. Recrystallization from n-hexane gave colorless blocks (75 mg).

### Refinement

H atoms on the C of methyl, methylene, methine and oxygen were positioned geomatrically with C–H=0.98–1.00 Å and O–H= 0.96 Å, respectively and constrained to ride on their parent atoms with *U*_iso_(H)= 1.2*U*_eq_(CH_2_, CH) and 1.5*U*_eq_(CH_3_, OH). A rotating group model was applied to the methyl groups. The absolute configuration (C3 R, C5 R, C10 S, C13 S, C14 R, C17 S, C20 S) was obtained by refining the Flack (1983) parameter to -0.35 (19).

### Crystal data

**Table:**

C_30_H_48_O_3_	*D*_x_ = 1.125 Mg m^−^^3^
*M**_r_* = 456.68	Cu *K*α radiation, λ = 1.54178 Å
Trigonal, *P*3_1_21	Cell parameters from 16991 reflections
*a* = 11.2794 (2) Å	θ = 3.6–71.8°
*c* = 36.6986 (6) Å	µ = 0.54 mm^−^^1^
*V* = 4043.45 (12) Å^3^	*T* = 100 K
*Z* = 6	Block, colorless
*F*(000) = 1512	0.29 × 0.24 × 0.13 mm

### Data collection

**Table:**

Bruker SMART APEXII DUO CCD diffractometer	5154 independent reflections
Radiation source: fine-focus sealed tube	5062 reflections with *I* > 2σ(*I*)
graphite	*R*_int_ = 0.040
φ and ω scans	θ_max_ = 71.8°, θ_min_ = 3.6°
Absorption correction: multi-scan (*SADABS*; Bruker, 2009)	*h* = −12→10
*T*_min_ = 0.859, *T*_max_ = 0.935	*k* = −13→13
29022 measured reflections	*l* = −45→43

### Refinement

**Table:**

Refinement on *F*^2^	Secondary atom site location: difference Fourier map
Least-squares matrix: full	Hydrogen site location: inferred from neighbouring sites
*R*[*F*^2^ > 2σ(*F*^2^)] = 0.038	H-atom parameters constrained
*wR*(*F*^2^) = 0.104	*w* = 1/[σ^2^(*F*_o_^2^) + (0.0607*P*)^2^ + 1.1875*P*] where *P* = (*F*_o_^2^ + 2*F*_c_^2^)/3
*S* = 0.97	(Δ/σ)_max_ < 0.001
5154 reflections	Δρ_max_ = 0.34 e Å^−^^3^
305 parameters	Δρ_min_ = −0.14 e Å^−^^3^
0 restraints	Absolute structure: Flack (1983), 2099 Friedel pairs
Primary atom site location: structure-invariant direct methods	Flack parameter: −0.35 (19)

### Special details

**Table:**

Experimental. The crystal was placed in the cold stream of an Oxford Cryosystems Cobra open-flow nitrogen cryostat (Cosier & Glazer, 1986) operating at 100.0 (1) K.
Geometry. All e.s.d.'s (except the e.s.d. in the dihedral angle between two l.s. planes) are estimated using the full covariance matrix. The cell e.s.d.'s are taken into account individually in the estimation of e.s.d.'s in distances, angles and torsion angles; correlations between e.s.d.'s in cell parameters are only used when they are defined by crystal symmetry. An approximate (isotropic) treatment of cell e.s.d.'s is used for estimating e.s.d.'s involving l.s. planes.
Refinement. Refinement of *F*^2^ against ALL reflections. The weighted *R*-factor *wR* and goodness of fit *S* are based on *F*^2^, conventional *R*-factors *R* are based on *F*, with *F* set to zero for negative *F*^2^. The threshold expression of *F*^2^ > σ(*F*^2^) is used only for calculating *R*-factors(gt) *etc*. and is not relevant to the choice of reflections for refinement. *R*-factors based on *F*^2^ are statistically about twice as large as those based on *F*, and *R*- factors based on ALL data will be even larger.

### Fractional atomic coordinates and isotropic or equivalent isotropic displacement parameters (Å^2^)

**Table:**

	*x*	*y*	*z*	*U*_iso_*/*U*_eq_	
O1	0.12186 (11)	0.02017 (12)	0.12824 (3)	0.0298 (2)	
H1O1	0.1165	0.0104	0.1543	0.045*	
O2	−0.10300 (11)	−0.06580 (12)	0.13280 (3)	0.0294 (2)	
O3	0.65407 (12)	0.90621 (15)	0.10227 (3)	0.0431 (3)	
H1O3	0.7207	0.9089	0.1129	0.065*	
C1	0.41649 (17)	0.72932 (17)	0.14749 (4)	0.0300 (3)	
H1A	0.3776	0.6804	0.1707	0.036*	
H1B	0.4653	0.6870	0.1357	0.036*	
C2	0.51897 (18)	0.87967 (18)	0.15572 (4)	0.0330 (3)	
H2A	0.4719	0.9207	0.1691	0.040*	
H2B	0.5926	0.8852	0.1716	0.040*	
C3	0.58166 (15)	0.96055 (17)	0.12096 (4)	0.0300 (3)	
H3A	0.6466	1.0583	0.1274	0.036*	
C4	0.47387 (16)	0.95453 (17)	0.09408 (4)	0.0314 (3)	
C5	0.36344 (15)	0.80196 (16)	0.08805 (4)	0.0276 (3)	
H5A	0.4118	0.7604	0.0751	0.033*	
C6	0.24806 (18)	0.78286 (18)	0.06230 (5)	0.0368 (4)	
H6A	0.1839	0.8038	0.0753	0.044*	
H6B	0.2869	0.8465	0.0414	0.044*	
C7	0.17160 (16)	0.63491 (17)	0.04855 (4)	0.0305 (3)	
H7A	0.0803	0.6141	0.0396	0.037*	
H7B	0.2224	0.6269	0.0276	0.037*	
C8	0.15280 (15)	0.52984 (16)	0.07647 (4)	0.0273 (3)	
C9	0.21172 (16)	0.56210 (16)	0.10983 (4)	0.0293 (3)	
C10	0.29897 (15)	0.71123 (16)	0.12250 (4)	0.0266 (3)	
C11	0.18977 (18)	0.45252 (16)	0.13711 (4)	0.0305 (3)	
H11A	0.1557	0.4717	0.1600	0.037*	
H11B	0.2801	0.4622	0.1426	0.037*	
C12	0.09172 (17)	0.30243 (16)	0.12677 (4)	0.0289 (3)	
H12A	0.1200	0.2428	0.1393	0.035*	
H12B	−0.0022	0.2755	0.1348	0.035*	
C13	0.09242 (15)	0.28248 (15)	0.08549 (4)	0.0248 (3)	
C14	0.05170 (15)	0.38169 (16)	0.06713 (4)	0.0261 (3)	
C15	0.03784 (19)	0.33660 (18)	0.02702 (4)	0.0343 (4)	
H15A	0.1284	0.3796	0.0149	0.041*	
H15B	−0.0227	0.3613	0.0135	0.041*	
C16	−0.02608 (18)	0.17853 (17)	0.02912 (4)	0.0328 (3)	
H16A	0.0254	0.1486	0.0134	0.039*	
H16B	−0.1226	0.1322	0.0209	0.039*	
C17	−0.01783 (15)	0.14260 (16)	0.06977 (4)	0.0256 (3)	
H17A	−0.1070	0.1187	0.0815	0.031*	
C18	0.23593 (16)	0.31412 (18)	0.07350 (4)	0.0328 (3)	
H18A	0.2655	0.2621	0.0886	0.049*	
H18B	0.3005	0.4123	0.0763	0.049*	
H18C	0.2332	0.2882	0.0479	0.049*	
C19	0.20263 (18)	0.7441 (2)	0.14522 (5)	0.0401 (4)	
H19A	0.1593	0.6757	0.1646	0.060*	
H19B	0.1319	0.7422	0.1293	0.060*	
H19C	0.2560	0.8353	0.1561	0.060*	
C20	0.00323 (16)	0.01791 (16)	0.07414 (4)	0.0263 (3)	
H20A	0.0943	0.0414	0.0637	0.032*	
C21	0.00184 (15)	−0.01382 (16)	0.11427 (4)	0.0253 (3)	
C22	−0.10897 (16)	−0.10907 (16)	0.05432 (4)	0.0273 (3)	
H22A	−0.1985	−0.1361	0.0657	0.033*	
H22B	−0.1133	−0.0848	0.0286	0.033*	
C23	−0.08548 (18)	−0.23138 (18)	0.05517 (5)	0.0339 (3)	
H23A	0.0057	−0.2034	0.0447	0.041*	
H23B	−0.0852	−0.2583	0.0808	0.041*	
C24	−0.19244 (19)	−0.35336 (18)	0.03441 (4)	0.0331 (3)	
H24A	−0.1793	−0.3524	0.0088	0.040*	
C25	−0.30231 (19)	−0.46146 (18)	0.04752 (4)	0.0353 (4)	
C26	−0.3398 (2)	−0.4808 (3)	0.08726 (5)	0.0611 (6)	
H26A	−0.2694	−0.4035	0.1012	0.092*	
H26B	−0.3461	−0.5660	0.0959	0.092*	
H26C	−0.4283	−0.4856	0.0906	0.092*	
C27	−0.4008 (2)	−0.5768 (2)	0.02355 (6)	0.0502 (5)	
H27A	−0.3706	−0.5563	−0.0019	0.075*	
H27B	−0.4923	−0.5877	0.0259	0.075*	
H27C	−0.4039	−0.6618	0.0310	0.075*	
C28	0.54466 (19)	1.0165 (2)	0.05767 (5)	0.0461 (5)	
H28A	0.6284	1.1039	0.0621	0.069*	
H28B	0.4828	1.0316	0.0421	0.069*	
H28C	0.5680	0.9534	0.0455	0.069*	
C29	0.4192 (2)	1.04643 (19)	0.10886 (6)	0.0443 (4)	
H29A	0.4931	1.1421	0.1088	0.066*	
H29B	0.3858	1.0187	0.1338	0.066*	
H29C	0.3441	1.0367	0.0933	0.066*	
C30	−0.08957 (17)	0.35873 (17)	0.07938 (5)	0.0346 (4)	
H30A	−0.1093	0.4232	0.0666	0.052*	
H30B	−0.0884	0.3737	0.1057	0.052*	
H30C	−0.1605	0.2648	0.0736	0.052*	

### Atomic displacement parameters (Å^2^)

**Table:**

	*U*^11^	*U*^22^	*U*^33^	*U*^12^	*U*^13^	*U*^23^
O1	0.0283 (5)	0.0419 (6)	0.0230 (5)	0.0203 (5)	−0.0021 (4)	−0.0007 (4)
O2	0.0250 (5)	0.0409 (6)	0.0199 (4)	0.0146 (5)	−0.0005 (4)	0.0017 (4)
O3	0.0251 (6)	0.0665 (9)	0.0380 (6)	0.0232 (6)	−0.0050 (5)	−0.0111 (6)
C1	0.0328 (8)	0.0353 (8)	0.0182 (6)	0.0144 (7)	−0.0059 (6)	−0.0014 (6)
C2	0.0327 (8)	0.0378 (9)	0.0218 (7)	0.0127 (7)	−0.0062 (6)	−0.0057 (6)
C3	0.0222 (7)	0.0365 (8)	0.0251 (7)	0.0099 (6)	−0.0041 (6)	−0.0042 (6)
C4	0.0229 (7)	0.0325 (8)	0.0305 (7)	0.0076 (7)	−0.0057 (6)	0.0028 (6)
C5	0.0218 (7)	0.0295 (8)	0.0242 (6)	0.0072 (6)	−0.0042 (5)	0.0037 (6)
C6	0.0283 (8)	0.0328 (9)	0.0374 (8)	0.0064 (7)	−0.0098 (7)	0.0096 (7)
C7	0.0249 (7)	0.0347 (8)	0.0230 (6)	0.0084 (6)	−0.0053 (6)	0.0061 (6)
C8	0.0209 (7)	0.0302 (8)	0.0254 (7)	0.0087 (6)	−0.0014 (5)	0.0055 (6)
C9	0.0287 (8)	0.0312 (8)	0.0220 (6)	0.0105 (6)	−0.0021 (6)	0.0042 (6)
C10	0.0225 (7)	0.0302 (8)	0.0239 (6)	0.0109 (6)	−0.0013 (6)	0.0032 (6)
C11	0.0377 (9)	0.0310 (8)	0.0190 (6)	0.0143 (7)	−0.0015 (6)	0.0013 (6)
C12	0.0347 (8)	0.0301 (8)	0.0183 (7)	0.0134 (7)	−0.0020 (6)	0.0029 (5)
C13	0.0220 (7)	0.0303 (7)	0.0195 (6)	0.0111 (6)	0.0004 (5)	0.0038 (5)
C14	0.0235 (7)	0.0306 (8)	0.0192 (6)	0.0099 (6)	−0.0025 (5)	0.0038 (6)
C15	0.0419 (9)	0.0345 (9)	0.0199 (7)	0.0141 (7)	−0.0068 (6)	0.0016 (6)
C16	0.0407 (9)	0.0358 (9)	0.0194 (7)	0.0172 (7)	−0.0057 (6)	−0.0002 (6)
C17	0.0243 (7)	0.0324 (8)	0.0184 (6)	0.0129 (6)	−0.0006 (5)	0.0004 (5)
C18	0.0236 (7)	0.0394 (9)	0.0321 (8)	0.0133 (7)	0.0016 (6)	0.0028 (7)
C19	0.0320 (8)	0.0386 (9)	0.0454 (9)	0.0143 (7)	0.0094 (7)	0.0034 (8)
C20	0.0256 (7)	0.0335 (8)	0.0195 (6)	0.0145 (6)	0.0005 (5)	0.0003 (6)
C21	0.0256 (7)	0.0292 (8)	0.0224 (6)	0.0148 (6)	−0.0021 (5)	−0.0018 (6)
C22	0.0313 (8)	0.0343 (8)	0.0179 (6)	0.0177 (7)	−0.0018 (5)	−0.0019 (6)
C23	0.0355 (8)	0.0376 (9)	0.0331 (8)	0.0216 (7)	−0.0006 (7)	0.0003 (7)
C24	0.0449 (9)	0.0356 (8)	0.0255 (7)	0.0251 (8)	0.0015 (7)	−0.0013 (6)
C25	0.0400 (9)	0.0388 (9)	0.0309 (8)	0.0224 (8)	−0.0010 (7)	0.0012 (7)
C26	0.0495 (12)	0.0782 (16)	0.0365 (10)	0.0176 (12)	0.0085 (9)	0.0057 (10)
C27	0.0547 (12)	0.0410 (10)	0.0492 (10)	0.0196 (10)	−0.0079 (9)	−0.0037 (8)
C28	0.0343 (9)	0.0401 (10)	0.0347 (8)	−0.0033 (8)	−0.0102 (7)	0.0079 (7)
C29	0.0350 (9)	0.0337 (9)	0.0607 (11)	0.0145 (7)	−0.0089 (8)	−0.0001 (8)
C30	0.0245 (7)	0.0285 (8)	0.0465 (9)	0.0101 (6)	−0.0018 (7)	0.0053 (7)

### Geometric parameters (Å, °)

**Table:**

O1—C21	1.3128 (18)	C15—C16	1.555 (2)
O1—H1O1	0.9626	C15—H15A	0.9900
O2—C21	1.2293 (18)	C15—H15B	0.9900
O3—C3	1.419 (2)	C16—C17	1.5606 (19)
O3—H1O3	0.8340	C16—H16A	0.9900
C1—C2	1.531 (2)	C16—H16B	0.9900
C1—C10	1.539 (2)	C17—C20	1.547 (2)
C1—H1A	0.9900	C17—H17A	1.0000
C1—H1B	0.9900	C18—H18A	0.9800
C2—C3	1.521 (2)	C18—H18B	0.9800
C2—H2A	0.9900	C18—H18C	0.9800
C2—H2B	0.9900	C19—H19A	0.9800
C3—C4	1.541 (2)	C19—H19B	0.9800
C3—H3A	1.0000	C19—H19C	0.9800
C4—C28	1.534 (2)	C20—C21	1.5139 (18)
C4—C29	1.545 (3)	C20—C22	1.539 (2)
C4—C5	1.555 (2)	C20—H20A	1.0000
C5—C6	1.534 (2)	C22—C23	1.530 (2)
C5—C10	1.5591 (19)	C22—H22A	0.9900
C5—H5A	1.0000	C22—H22B	0.9900
C6—C7	1.531 (2)	C23—C24	1.506 (2)
C6—H6A	0.9900	C23—H23A	0.9900
C6—H6B	0.9900	C23—H23B	0.9900
C7—C8	1.499 (2)	C24—C25	1.320 (3)
C7—H7A	0.9900	C24—H24A	0.9500
C7—H7B	0.9900	C25—C27	1.502 (3)
C8—C9	1.353 (2)	C25—C26	1.504 (2)
C8—C14	1.518 (2)	C26—H26A	0.9800
C9—C11	1.512 (2)	C26—H26B	0.9800
C9—C10	1.536 (2)	C26—H26C	0.9800
C10—C19	1.555 (2)	C27—H27A	0.9800
C11—C12	1.537 (2)	C27—H27B	0.9800
C11—H11A	0.9900	C27—H27C	0.9800
C11—H11B	0.9900	C28—H28A	0.9800
C12—C13	1.5319 (18)	C28—H28B	0.9800
C12—H12A	0.9900	C28—H28C	0.9800
C12—H12B	0.9900	C29—H29A	0.9800
C13—C18	1.537 (2)	C29—H29B	0.9800
C13—C17	1.551 (2)	C29—H29C	0.9800
C13—C14	1.559 (2)	C30—H30A	0.9800
C14—C15	1.539 (2)	C30—H30B	0.9800
C14—C30	1.548 (2)	C30—H30C	0.9800
			
C21—O1—H1O1	111.0	C16—C15—H15B	110.9
C3—O3—H1O3	117.4	H15A—C15—H15B	109.0
C2—C1—C10	112.80 (14)	C15—C16—C17	106.94 (12)
C2—C1—H1A	109.0	C15—C16—H16A	110.3
C10—C1—H1A	109.0	C17—C16—H16A	110.3
C2—C1—H1B	109.0	C15—C16—H16B	110.3
C10—C1—H1B	109.0	C17—C16—H16B	110.3
H1A—C1—H1B	107.8	H16A—C16—H16B	108.6
C3—C2—C1	111.47 (12)	C20—C17—C13	118.46 (12)
C3—C2—H2A	109.3	C20—C17—C16	113.03 (12)
C1—C2—H2A	109.3	C13—C17—C16	102.30 (12)
C3—C2—H2B	109.3	C20—C17—H17A	107.5
C1—C2—H2B	109.3	C13—C17—H17A	107.5
H2A—C2—H2B	108.0	C16—C17—H17A	107.5
O3—C3—C2	109.98 (14)	C13—C18—H18A	109.5
O3—C3—C4	106.45 (12)	C13—C18—H18B	109.5
C2—C3—C4	112.99 (13)	H18A—C18—H18B	109.5
O3—C3—H3A	109.1	C13—C18—H18C	109.5
C2—C3—H3A	109.1	H18A—C18—H18C	109.5
C4—C3—H3A	109.1	H18B—C18—H18C	109.5
C28—C4—C3	108.55 (13)	C10—C19—H19A	109.5
C28—C4—C29	106.96 (16)	C10—C19—H19B	109.5
C3—C4—C29	108.45 (14)	H19A—C19—H19B	109.5
C28—C4—C5	109.05 (13)	C10—C19—H19C	109.5
C3—C4—C5	108.52 (13)	H19A—C19—H19C	109.5
C29—C4—C5	115.14 (14)	H19B—C19—H19C	109.5
C6—C5—C4	113.39 (13)	C21—C20—C22	109.46 (12)
C6—C5—C10	108.84 (12)	C21—C20—C17	109.12 (12)
C4—C5—C10	117.61 (12)	C22—C20—C17	111.61 (12)
C6—C5—H5A	105.3	C21—C20—H20A	108.9
C4—C5—H5A	105.3	C22—C20—H20A	108.9
C10—C5—H5A	105.3	C17—C20—H20A	108.9
C7—C6—C5	109.39 (14)	O2—C21—O1	121.94 (12)
C7—C6—H6A	109.8	O2—C21—C20	122.99 (13)
C5—C6—H6A	109.8	O1—C21—C20	115.06 (12)
C7—C6—H6B	109.8	C23—C22—C20	113.39 (13)
C5—C6—H6B	109.8	C23—C22—H22A	108.9
H6A—C6—H6B	108.2	C20—C22—H22A	108.9
C8—C7—C6	114.61 (13)	C23—C22—H22B	108.9
C8—C7—H7A	108.6	C20—C22—H22B	108.9
C6—C7—H7A	108.6	H22A—C22—H22B	107.7
C8—C7—H7B	108.6	C24—C23—C22	113.12 (14)
C6—C7—H7B	108.6	C24—C23—H23A	109.0
H7A—C7—H7B	107.6	C22—C23—H23A	109.0
C9—C8—C7	123.32 (14)	C24—C23—H23B	109.0
C9—C8—C14	119.87 (13)	C22—C23—H23B	109.0
C7—C8—C14	116.57 (12)	H23A—C23—H23B	107.8
C8—C9—C11	121.45 (14)	C25—C24—C23	127.76 (14)
C8—C9—C10	121.88 (13)	C25—C24—H24A	116.1
C11—C9—C10	116.62 (12)	C23—C24—H24A	116.1
C9—C10—C1	111.30 (13)	C24—C25—C27	122.20 (16)
C9—C10—C19	106.40 (13)	C24—C25—C26	123.61 (17)
C1—C10—C19	107.89 (13)	C27—C25—C26	114.19 (17)
C9—C10—C5	107.84 (12)	C25—C26—H26A	109.5
C1—C10—C5	107.95 (12)	C25—C26—H26B	109.5
C19—C10—C5	115.50 (13)	H26A—C26—H26B	109.5
C9—C11—C12	118.07 (12)	C25—C26—H26C	109.5
C9—C11—H11A	107.8	H26A—C26—H26C	109.5
C12—C11—H11A	107.8	H26B—C26—H26C	109.5
C9—C11—H11B	107.8	C25—C27—H27A	109.5
C12—C11—H11B	107.8	C25—C27—H27B	109.5
H11A—C11—H11B	107.1	H27A—C27—H27B	109.5
C13—C12—C11	110.63 (12)	C25—C27—H27C	109.5
C13—C12—H12A	109.5	H27A—C27—H27C	109.5
C11—C12—H12A	109.5	H27B—C27—H27C	109.5
C13—C12—H12B	109.5	C4—C28—H28A	109.5
C11—C12—H12B	109.5	C4—C28—H28B	109.5
H12A—C12—H12B	108.1	H28A—C28—H28B	109.5
C12—C13—C18	109.33 (12)	C4—C28—H28C	109.5
C12—C13—C17	117.18 (11)	H28A—C28—H28C	109.5
C18—C13—C17	110.19 (13)	H28B—C28—H28C	109.5
C12—C13—C14	107.28 (12)	C4—C29—H29A	109.5
C18—C13—C14	111.32 (12)	C4—C29—H29B	109.5
C17—C13—C14	101.25 (11)	H29A—C29—H29B	109.5
C8—C14—C15	118.41 (13)	C4—C29—H29C	109.5
C8—C14—C30	105.21 (13)	H29A—C29—H29C	109.5
C15—C14—C30	107.25 (13)	H29B—C29—H29C	109.5
C8—C14—C13	111.32 (12)	C14—C30—H30A	109.5
C15—C14—C13	101.18 (12)	C14—C30—H30B	109.5
C30—C14—C13	113.73 (12)	H30A—C30—H30B	109.5
C14—C15—C16	104.10 (12)	C14—C30—H30C	109.5
C14—C15—H15A	110.9	H30A—C30—H30C	109.5
C16—C15—H15A	110.9	H30B—C30—H30C	109.5
C14—C15—H15B	110.9		
			
C10—C1—C2—C3	−58.31 (18)	C11—C12—C13—C17	−170.39 (13)
C1—C2—C3—O3	−61.29 (17)	C11—C12—C13—C14	−57.48 (16)
C1—C2—C3—C4	57.51 (19)	C9—C8—C14—C15	−151.76 (16)
O3—C3—C4—C28	−49.08 (18)	C7—C8—C14—C15	33.7 (2)
C2—C3—C4—C28	−169.90 (15)	C9—C8—C14—C30	88.46 (17)
O3—C3—C4—C29	−164.95 (14)	C7—C8—C14—C30	−86.04 (16)
C2—C3—C4—C29	74.23 (17)	C9—C8—C14—C13	−35.2 (2)
O3—C3—C4—C5	69.31 (16)	C7—C8—C14—C13	150.34 (13)
C2—C3—C4—C5	−51.51 (18)	C12—C13—C14—C8	60.59 (15)
C28—C4—C5—C6	−63.26 (19)	C18—C13—C14—C8	−58.98 (16)
C3—C4—C5—C6	178.67 (14)	C17—C13—C14—C8	−176.07 (11)
C29—C4—C5—C6	56.93 (19)	C12—C13—C14—C15	−172.69 (12)
C28—C4—C5—C10	168.18 (15)	C18—C13—C14—C15	67.73 (15)
C3—C4—C5—C10	50.10 (18)	C17—C13—C14—C15	−49.35 (14)
C29—C4—C5—C10	−71.63 (18)	C12—C13—C14—C30	−58.04 (16)
C4—C5—C6—C7	162.20 (13)	C18—C13—C14—C30	−177.61 (13)
C10—C5—C6—C7	−64.86 (17)	C17—C13—C14—C30	65.30 (15)
C5—C6—C7—C8	38.2 (2)	C8—C14—C15—C16	159.15 (14)
C6—C7—C8—C9	−7.5 (2)	C30—C14—C15—C16	−82.13 (15)
C6—C7—C8—C14	166.80 (14)	C13—C14—C15—C16	37.27 (15)
C7—C8—C9—C11	179.99 (15)	C14—C15—C16—C17	−11.73 (17)
C14—C8—C9—C11	5.9 (2)	C12—C13—C17—C20	−77.28 (17)
C7—C8—C9—C10	2.6 (2)	C18—C13—C17—C20	48.55 (16)
C14—C8—C9—C10	−171.46 (14)	C14—C13—C17—C20	166.46 (12)
C8—C9—C10—C1	−146.20 (15)	C12—C13—C17—C16	157.72 (13)
C11—C9—C10—C1	36.34 (18)	C18—C13—C17—C16	−76.45 (14)
C8—C9—C10—C19	96.51 (18)	C14—C13—C17—C16	41.45 (14)
C11—C9—C10—C19	−80.96 (17)	C15—C16—C17—C20	−147.10 (14)
C8—C9—C10—C5	−28.0 (2)	C15—C16—C17—C13	−18.60 (16)
C11—C9—C10—C5	154.56 (14)	C13—C17—C20—C21	63.61 (16)
C2—C1—C10—C9	170.77 (12)	C16—C17—C20—C21	−176.81 (13)
C2—C1—C10—C19	−72.85 (16)	C13—C17—C20—C22	−175.29 (11)
C2—C1—C10—C5	52.61 (17)	C16—C17—C20—C22	−55.71 (17)
C6—C5—C10—C9	58.30 (17)	C22—C20—C21—O2	−49.73 (19)
C4—C5—C10—C9	−171.02 (13)	C17—C20—C21—O2	72.68 (18)
C6—C5—C10—C1	178.66 (14)	C22—C20—C21—O1	131.25 (14)
C4—C5—C10—C1	−50.65 (18)	C17—C20—C21—O1	−106.34 (15)
C6—C5—C10—C19	−60.53 (18)	C21—C20—C22—C23	−64.01 (16)
C4—C5—C10—C19	70.16 (18)	C17—C20—C22—C23	175.09 (13)
C8—C9—C11—C12	−3.3 (2)	C20—C22—C23—C24	−177.61 (12)
C10—C9—C11—C12	174.14 (14)	C22—C23—C24—C25	−97.4 (2)
C9—C11—C12—C13	30.5 (2)	C23—C24—C25—C27	−179.74 (18)
C11—C12—C13—C18	63.36 (17)	C23—C24—C25—C26	−0.1 (3)

### Hydrogen-bond geometry (Å, °)

**Table:**

*D*—H···*A*	*D*—H	H···*A*	*D*···*A*	*D*—H···*A*
O1—H1O1···O2^i^	0.96	1.74	2.6762 (14)	162
O3—H1O3···O2^ii^	0.83	2.00	2.828 (2)	172
C12—H12A···O1	0.99	2.55	3.367 (2)	139
C22—H22A···O3^iii^	0.99	2.36	3.276 (2)	153

Symmetry codes: (i) −*x*, −*x*+*y*, −*z*+1/3; (ii) *x*+1, *y*+1, *z*; (iii) *x*−1, *y*−1, *z*.

## References

[bb1] Atawodi, S. E. (2010). *Adv. Biol. Res.* **4**, 314–322.

[bb2] Bruker (2009). *APEX2*, *SAINT* and *SADABS* Bruker AXS Inc., Madison, Wisconsin, USA.

[bb9] Cosier, J. & Glazer, A. M. (1986). *J. Appl. Cryst.* **19**, 105–107.

[bb3] Dongmo, P. M., Tchoumbougnang, F., Ndongson, B., Agwannande, W., Sandjon, B., Zollo, P. H. A. & Menut, C. (2010). *Agric. Biol. J. N. Am.* **1** 606–6011.

[bb4] Flack, H. D. (1983). *Acta Cryst.* A**39**, 876–881.

[bb5] Mora, A. J., Delgado, G., Díaz de Delgado, G., Usubillaga, A., Khouri, N. & Bahsas, A. (2001). *Acta Cryst.* C**57**, 638–640. 10.1107/s0108270101003316 11353277

[bb6] Sheldrick, G. M. (2008). *Acta Cryst.* A**64**, 112–122. 10.1107/S0108767307043930 18156677

[bb7] Spek, A. L. (2009). *Acta Cryst.* D**65**, 148–155. 10.1107/S090744490804362X PMC2631630 19171970

[bb8] Tchiégang, C., Kapchié, N. V., Kapseu, C. & Parmentier, M. (2001). *J. Food Eng.* **47**, 63–68.

